# State Boards of Health

**Published:** 1877-05

**Authors:** 


State Boards of Health.—We have received reports of the State Boards of Health of Georgia, Wisconsin, Minnesota, and Michigan. All of them contain sufficient to justify the existence of the Boards, but the Michigan report appears to be the best. Many of our readers may remember the health tracts of John Brown of Edinburgh. We would suggest that a very important work might be done by having distributed well-written sanitary tracts. The success of tracts in religious matters is thought to justify the expense; and certainly this method might be availed of in spreading a practical knowledge of hygiene. Would our tract societies aid?—*The Medical Times.*

